# Maximum dose, safety, tolerability and ketonemia after triheptanoin in glucose transporter type 1 deficiency (G1D)

**DOI:** 10.1038/s41598-023-30578-z

**Published:** 2023-03-01

**Authors:** Ignacio Málaga, Adrian Avila, Sharon Primeaux, Raja Reddy Kallem, Charles R. Roe, William C. Putnam, Jason Y. Park, Shlomo Shinnar, Chul Ahn, Juan M. Pascual

**Affiliations:** 1 Rare Brain Disorders Program, Department of Neurology, The University of Texas Southwestern Medical Center, 5323 Harry Hines Blvd. Mail Code 8813, Dallas, TX 75390 USA; 2 Department of Pharmacy Practice and Clinical Pharmacology, Experimental Therapeutics Center, Texas Tech University Health Sciences Center, Dallas, TX 75235 USA; 3 Department of Pharmaceutical Science, School of Pharmacy, Texas Tech University Health Sciences Center, Dallas, TX 75235 USA; 4 Department of Pathology, The University of Texas Southwestern Medical Center, Dallas, TX USA; 5 Departments of Neurology and Pediatrics, Albert Einstein College of Medicine, Bronx, NY 10467 USA; 6 Department of Population and Data Sciences, The University of Texas Southwestern Medical Center, Dallas, TX USA; 7 Department of Physiology, The University of Texas Southwestern Medical Center, Dallas, USA; 8 Department of Pediatrics, The University of Texas Southwestern Medical Center, Dallas, USA; 9 Eugene McDermott Center for Human Growth & Development/Center for Human Genetics, The University of Texas Southwestern Medical Center, Dallas, TX USA

**Keywords:** Paediatric neurological disorders, Phase I trials

## Abstract

Augmentation of anaplerosis, or replenishment of carbon lost during intermediary metabolic transitions, is desirable in energy metabolism defects. Triheptanoin, the triglyceride of 7-carbon heptanoic acid, is anaplerotic via direct oxidation or 5-carbon ketone body generation. In this context, triheptanoin can be used to treat Glucose transporter type 1 deficiency encephalopathy (G1D). An oral triheptanoin dose of 1 g/Kg/day supplies near 35% of the total caloric intake and impacted epilepsy and cognition in G1D. This provided the motivation to establish a maximum, potentially greater dose. Using a 3 + 3 dose-finding approach useful in oncology, we studied three age groups: 4–6, 6.8–10 and 11–16 years old. This allowed us to arrive at a maximum tolerated dose of 45% of daily caloric intake for each group. Safety was ascertained via analytical blood measures. One dose-limiting toxicity, occurring in 1 of 6 subjects, was encountered in the middle age group in the context of frequently reduced gastrointestinal tolerance for all groups. Ketonemia following triheptanoin was determined in another group of G1D subjects. In them, β-ketopentanoate and β-hydroxypentanoate concentrations were robustly but variably increased. These results enable the rigorous clinical investigation of triheptanoin in G1D by providing dosing and initial tolerability, safety and ketonemic potential.

*ClinicalTrials.gov registration*: NCT03041363, first registration 02/02/2017.

## Introduction

In normal conditions, carbon enters the organism with the diet and exits via excretion as organic waste or respiration as CO_2_. The principal source of CO_2_ is cellular respiration, which comprises the full oxidation of glucose-derived pyruvate in the tricarboxylic acid (TCA) cycle. At completion, pyruvate oxidation via pyruvate dehydrogenase, together with other catabolic reactions, is associated with net carbon loss or negative carbon balance^1^. This is in part counteracted by the parallel carboxylation of a fraction of pyruvate, which replenishes TCA cycle intermediate substrates. This, and other carbon-refilling reactions, are collectively termed anaplerosis^2^. Both pyruvate oxidation and decarboxylation are essential to sustain the robust metabolic rate that characterizes neural tissue. Despite wide variation attributable to measurement methods, a significant fraction of brain glucose flux, perhaps amounting to 20%, is estimated to sustain anaplerosis^2,3^. Similarly to pyruvate dehydrogenase, whose deficiency leads preferentially to encephalopathy^4^, the relevance of pyruvate carboxylation and anaplerosis to brain function is underscored by the human disease pyruvate carboxylase deficiency, where even mild reductions in enzyme activity are associated with neural dysfunction^5^. Intermediary carbon depletion has also been documented in more common disorders such as epilepsy^6^. Thus, extending this reasoning to the preceding metabolic events, the availability of pyruvate-derived byproducts is also likely to be reduced in clinically relevant states associated with diminished brain glucose transport into and flux through the brain. An example of such a state is Glucose transporter deficiency type 1 (G1D), where decreased blood to brain and glial cell glucose transport^7^ is associated with encephalopathy^8^.

G1D constitutes primarily a neurological disorder without alteration of systemic metabolism^9,10^. Some of its manifestations prove fully or partially treatable with a ketogenic diet in a significant fraction of individuals^11,12^. Nevertheless, the diet’s main ketone byproducts, β-hydroxybutyrate and acetoacetate, are not anaplerotic. Further, the diet is also poor in carbohydrate and is thus associated with a reduced blood glucose concentration. This is counterintuitive in G1D because all individuals to date have been haploinsufficient (mouse null *SLC2A1* mutations are embryonic lethal^13^) and thus possess substantial glucose transport capacity, and many respond favorably to elevated blood glucose^7^. Moreover, the ketogenic diet is associated with side effect risks^14–18^. These notions provide the motivation to study anaplerotic therapy for G1D.

One biochemical principle sustains this motivation, since an alternative anaplerotic route of entry into the TCA cycle is provided by the reaction catalyzed by propionyl coenzyme A carboxylase. This reaction enables the utilization of exogenously administered odd-carbon-number containing fatty acids such as heptanoate and its metabolic byproducts as substrates for anaplerosis. Used as a supplement to a regular diet, triheptanoin (C7), the triglyceride of heptanoate, is associated with favorable impact on several aspects of human G1D encephalopathy^19^.

Previous studies suggest that C7 dosed at up to 35% daily caloric intake causes no discernible abnormalities in normal individuals and may be beneficial in inherited lipid or carbohydrate metabolic disorders^19–23^. To discern if a greater dose is feasible in G1D children, we used a 3 + 3 dose-finding design adapted from oncological phase I clinical trials^24,25^. Our objectives were to determine the safety and tolerability of C7, its maximum tolerated dose (MTD) and its ketogenic potential via the formation of the C5 ketones β-ketopentanoate (BKP) and β-hydroxypentanoate (BHP).

## Materials and methods

We followed the ethical standards of the Helsinki Declaration of 1975 (as revised in 1983) and was approved by the Institutional Review Board approval of UT Southwestern Medical Center. Informed consent was obtained in writing from all the subjects or legally authorized representatives. Assent was also equally documented for cognitively-capable children between 10 and 17 years of age.

We utilized sets of data from three independent groups of G1D individuals prospectively studied at the Rare Brain Disorders Program of the University of Texas Southwestern Medical Center: (1) 14 subjects (ages 2–27 years, group A) who consumed C7 at a 35% caloric dose and were previously reported as part of the first study of triheptanoin in G1D^19^ (ClinicalTrials.gov Identifier NCT02018315, first registration 23/12/2013). No dose-finding was part of that study, since the 35% dose was chosen based only on the dose used in other unrelated disorders. However, a new analysis of these data is used here for initial dose tolerability estimation and the data are therefore presented as newly separated by age group. This was not previously reported. As such, only the information relevant to the present work is contained in a table and the age dependence of tolerability discussed; (2) 12 subjects (ages 4–16 years, group B) enrolled in a 3 + 3 dose-finding phase I clinical trial (NCT03041363, first registration 02/02/2017). These subjects were studied between March and December of 2017 and their data were intended solely for the determination of the MTD and associated safety and tolerability; (3) 7 individuals (ages 2–11 years, group C) who received analytical testing at the MTD, once it was determined using the knowledge gained from group A and group B. These subjects were studied between January 2018 and December 2020 (ClinicalTrials.gov Identifier NCT03181399, first registration 08/06/2017). They received evaluation of ketonemia and additional safety assessments and these data are used in the present work. These subjects then proceeded to a still ongoing, different study unrelated to the goals of the present work, and this is therefore not further described here.

### Participant characteristics

The diagnosis was ascertained via DNA analysis of the *SLC2A1* gene, which encodes Glut1, or via fluoro-deoxyglucose positron emission tomography (PET) of the brain when DNA results did not reveal a probable pathogenic variant^7,9,26^. The DNA variants cited here refer to transcript NM_006516.2 and were deemed pathogenic as per each clinical DNA testing report. Enrolment followed the order of contact made by eligible subjects. Eligible contacts spontaneously exceeded enrolment targets. All individuals were consuming a regular or a modified Atkins diet. The latter type of diet was selected as it represents the most common alternative to a standard ketogenic diet^12^, thus facilitating enrolment in this study. There was no consideration of geographic location (U.S. or abroad) or disease severity. The phenotypic features of all subjects included a variable combination of intellectual disability, epilepsy, ataxia, or episodic apraxia. For the 3 + 3 study, medications, including antiseizure drugs, were not allowed to change 30 days prior to or during the study. Supplementary Table S1 lists ages, clinically significant DNA variants, phenotypes and previous treatments for the 14 group A subjects previously studied ^19^. Tables 1 and 2 indicate the same characteristics for groups A and B. Supplementary Table S2 lists the complete inclusion and exclusion criteria for group B.

**Table 1:** Characteristics of group B individuals studied for dose-finding.

Subject number	Age at enrollment (years), gender	Phenotype	*SLC2A1* variant	Previous treatments	Current treatment	Age group
B1	4, F	Epilepsy, ID	c.997 C > T (p.Arg333Trp)	ASM	None	Group BI
B2	6, F	Epilepsy, ID	c.209 C > T (p.Ala70Val)	MAD	None	Group BI
B3	16, F	Epilepsy, ID	c.982 G > T (p.Val328Leu)	KD	None	Group BIII
B4	9, F	Epilepsy, ID	c.1279–3 C > G Splice intron 9	KD	None	Group BII
B5	9, F	Epilepsy, ID, ataxia	c.290 T > C (p.Leu97Pro)	MAD	None	Group BII
B6	8, M	Epilepsy, ID, ataxia	c.737_740del (p.Glu246Glyfs*5)	KD, MAD	MAD	Group BII
B7	11, M	Epilepsy, ataxia	c.667 C > T (p.Arg223Trp)	MAD	MAD	Group BII
B8	16, F	Epilepsy, ID, ataxia	c.1189 C > T (p.Gln397*)	KD, MAD	MAD	Group BIII
B9	6, M	Epilepsy, ID, ataxia	c.1198 C > T (p.Arg400Cys)	KD, MAD	MAD	Group BI
B10	6, F	Epilepsy, ID, ataxia	c.1177 G > A (p.Glu393Lys)	KD	None	Group BII
B11	7, M	Apraxia, ID	c.1034 C > T (p.Ala345Val)	None	None	Group BII
B12	10, F	Epilepsy, ID, ataxia, apraxia	PET positive	KD, MAD	None	Group BIII

Demographic data, phenotype, *SLC2A1* variant (using reference transcript NM_006516.2) and dietary or pharmacologic treatments received throughout life and current treatment at enrolment. *M* male, *F* female, *ID* intellectual disability, *KD* Ketogenic diet, *MAD* Modified Atkins diet, *ASM* antiseizure medications, *PET* *positive* diagnosed by fluoro-deoxyglucose PET study.

**Table 2:** Characteristics of group C individuals studied for ketonemia.

Subject number	Age at enrollment (years), gender	Phenotype	*SLC2A1* variant	Previous treatments	Current treatment
C1	6, M	Epilepsy, ID	c.880 T > C (p.Ser294Pro)	MAD	MAD
C2	11, F	Epilepsy, apraxia, ID	c.940 G > A (p.Gly314Ser)	MAD	MAD
C3	6, M	Epilepsy, ID	c.940 G > A (p.Gly314Ser)	KD	None
C4	3, F	Epilepsy, ID	c.1198 C > T (p.Arg400Cys)	ASM	None
C5	7, F	Epilepsy, ID	c.1199 G > A (p.Arg400His)	ASM	None
C6	7, F	Epilepsy, ID	PET positive	ASM	None
C7	2, F	Epilepsy, ID	c.1198 C > T (p.Arg400Cys)	KD	None

Demographic data, phenotype, *SLC2A1* variant and dietary or pharmacologic treatments received throughout life and current treatment at enrolment. *M* male, *F* female, *ID* intellectual disability, *KD* Ketogenic diet, *MAD* Modified Atkins diet, *ASM* antiseizure medications, *PET positive* diagnosed via fluoro-deoxyglucose PET study.

### Treatment protocol

#### MTD study

**Figure 1:**
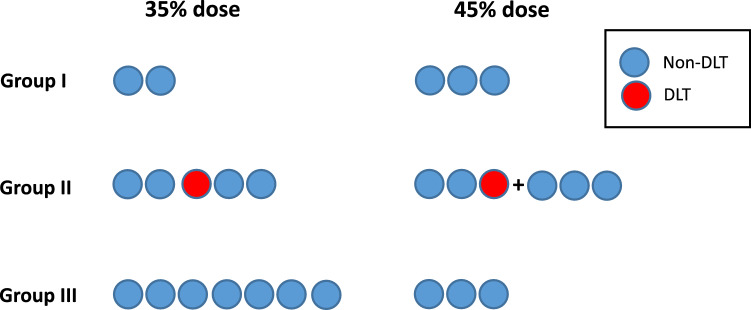
C7 dose-finding. Subjects are divided in 3 age groups as described in the methods. Each circle represents one subject. Left: 14 group A G1D individuals studied at the 35% dose^19^. One DLT was observed in age group AII. Right: Tolerance of the 45% dose in group B following the 3 + 3 design. The MTD was determined at 45% in groups BI and BIII after all 3 subjects in each group tolerated this dose. Due to one DTL in group BII, an additional 3 subjects were added to this group, following after tolerance, in a determination of the same MTD for this age group. *DLT* Dose limiting toxicity.

A standard 3 + 3 phase I dose de-escalation design was used in group B subjects. The MTD was defined as the dose at which fewer than one third of patients experienced a dose-limiting toxicity (DLT). As part of the study design, if dose 1 exceeded the MTD, the dose would be de-escalated in subsequent subjects to dose 2. If dose 2 proved intolerable, then 35% would be considered as the MTD based on the dose tolerated by group B^19^. Intra-patient dose escalation was permitted. The dose range under consideration (35–45%) was informed by nutritional standards^27,28^. Figure 1 illustrates the division of subjects by age: group BI (4–6 years old), group BII (6.8 to 10 years old) and group BIII (11–16 years old). Following a standard physical (including neurological) examination and a nutritional assessment to estimate average daily calories consumed, the subjects received baseline blood measurements (comprehensive metabolic panel, complete blood count, lipid panel, lactate, and β-hydroxybutyrate). C7 was then dosed for each subject as a percentage of the daily caloric intake (dose 1 = 45%; dose 2 = 40%), divided into 4 doses per day. To minimize insulin-mediated suppression of ketogenesis and potential competition of dietary octanoate with C7^29^, each dose was consumed 45–60 min before meals. A reduction in calories from other dietary sources compensated for the extra C7 calories. All subjects ingested C7 daily for 7 days. We measured tolerance over 7 days because we previously reported acceptable tolerance of the 35% dose after 2 days^30^. They received physical examination, tolerability and toxicity assessments every 2–3 days until exit, with additional telephone contact on non-physical examination days to assess side effects. Blood studies were repeated at physical examination visits. Upon discontinuation of C7 on day 8, the subjects received a physical examination, repeat blood studies and tolerability and toxicity assessment, all of which was again repeated on day 10. Side effects were again assessed one-month post-exit via telephone. Toxicity was assessed using the Common Toxicity Criteria (version 5.0) of the National Cancer Institute, National Institutes of Health. A dose limiting toxicity (DLT) was defined as any toxicity grade ≥ 3 (except for grade 3 nausea, vomiting, or diarrhea that could be controlled within 24 h with supportive care measures) or any unacceptable grade 2 toxicity resulting in treatment intolerance despite maximal medical treatment.

#### C7 ketonemia

**Figure 2:**
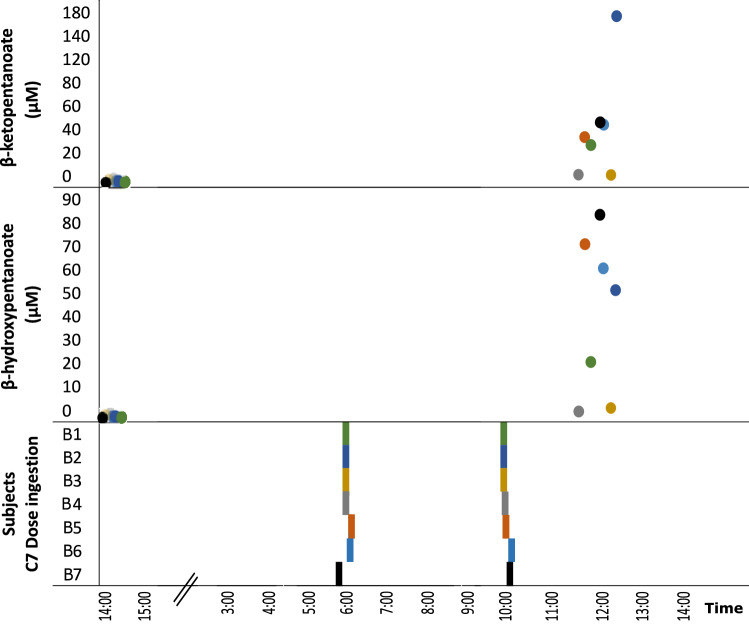
Stimulation of C5 ketogenesis by C7. Plasma levels of C5 ketones before and after two C7 doses. Top and middle panels illustrate C5 ketone values before and after an overnight fasting period followed by two C7 doses. The baseline (pre-C7) values were obtained at 2–2:30 PM. Two C7 doses (each ¼ of the MTD) were administered approximately at 6 AM and 10 AM. The only morning meal was breakfast approximately at 8 AM. Each color symbol and time identifies one individual.

Group C subjects received baseline analytical measurements (comprehensive metabolic panel, complete blood count, lipid panel, lactate, β-hydroxybutyrate, β-ketopentanoate and β-hydroxypentanoate) and physical examination. They then observed an overnight fast. C7 was calculated at a daily dose of 45% of daily caloric intake. The following morning, two doses were administered at approximately 6 AM (before breakfast) and 10 AM (before lunch), each containing ¼ of the total daily dose. Before lunch, at 12–1 PM, blood was collected for determination of ketone bodies as described^31^, approximately 21 h after the baseline analysis. Figure 2 illustrates this experimental sequence.

### C7 dietary supplement

Triheptanoin (glycerol triheptanoate, Neobee 795) was provided by Stepan Lipid Nutrition from a supply used in the human food industry as an additive to dairy products or as an emollient in cosmetics. Participants and caretakers were instructed on its mode of administration. To facilitate consumption approximately one hour before meals, in some cases the C7 oil was mixed immediately before use with a small amount (less than 15 ml) of fat free, sugar free yogurt, pudding, or an equivalent low-calorie food.

## Results

### Subject characteristics

Group B subjects, summarized in Table 1, included 8 females and 4 males. Median age was 8.5 years (range 4–16). They were primary English and Spanish speakers, all of whom identified themselves as white. Eight (67%) participants were non-Hispanic and 4 (33%) were Hispanic. One subject who exhibited common disease features and decreased cerebrospinal fluid glucose (25 mg/dl) was enrolled using the findings of a PET study, consistent with the approximately 15% of G1D patients who do not harbor an identifiable a pathogenic variant in *SLC2A1*^10^,

Group C subjects, studied for ketonemia, are summarized in Table 2. This group included 5 females and 2 males, with a median age of 8.5 years (range 4–16 years). It also included English and Spanish speakers. All of them identified themselves as white. Five participants identified as non-Hispanic, and two as Hispanic. One subject with no pathogenic mutation in *SLC2A1* exhibited characteristic symptoms and a brain PET study indicative of G1D.

### Adverse events

We previously collected safety data from group A subjects, comprising 265 G1D subject-months while receiving C7 treatment (^19^, with subsequent data collection until September 1 of 2015). These data originated from 14 G1D individuals (age 2–27 years) recruited from our program, who received C7 at a 35% dose. All received a regular diet adjusted to maintain the appropriate daily calories after the addition of C7. Of these, following study completion, 11 subjects opted to maintain longer term C7 consumption. There were no statistically significant differences in blood glucose, β-hydroxybutyrate, lactate, cholesterol (HDL, LDL, total), triglycerides, basic metabolic panel electrolytes, blood urea nitrogen, creatinine, creatine kinase, AST, ALT, GGT and blood cell count before and after C7 treatment. Three subjects experienced gastrointestinal discomfort within two days of initiation that subsided after decreasing the C7 dose, followed by a gradual increase to the desired dose over 7–14 days. One subject maintained pre-C7 levels of caloric consumption and gained 4 kg (baseline weight 52 kg) over 6 weeks, which were lost over another 6 weeks following nutritional counseling. There was no change in analytical parameters in this subject at maximum weight.

No subject in the current dose-finding and ketonemia studies experienced serious or unexpected adverse events. There were no appreciable physical examination changes in any of these subjects. Table 3 reflects the adverse effects and event noted in the dose-finding individuals. Eleven of the twelve subjects in the 3 + 3 study (group B) experienced mild adverse events. Seven (58%) had mild diarrhea or abdominal discomfort within 4 days of treatment initiation. Five (42%) experienced mild vomiting within 3 days of treatment initiation. In four of the five (33%), the digestive discomfort, diarrhea or vomiting symptoms resolved by reducing the amount of triheptanoin by one half and gradually titrating up the maximum target level over 3 days such that the 45% dose was considered tolerable. One subject exhibited persistent gastrointestinal intolerance. Therefore, following the 3 + 3 design, the middle age group was expanded by 3 more subjects to a total of 6, with the latter three exhibiting tolerance. One subject had a grand mal seizure during the study. The occurrence of this event was judged consistent with the previous frequency and severity of epilepsy in this subject. Group C subjects manifested no adverse events. All the participants reported baseline wellbeing one month after study completion.

**Table 3:** Adverse effects of C7 and event during the study.

	Grade 1–2 *n* (%)	Grade 3 *n* (%)
Adverse effects		
Emesis	5 (60%)	1 (8%)
Nausea	1 (8%)	
Diarrhea	6 (50%)	
Abdominal discomfort	2 (17%)	1 (8%)
Neurological event		
Seizure	1 (8%)	

Effects in group B subjects were graded from 1 to 3 using Common Toxicity Criteria. N: number of subjects. The seizure was judged unrelated to C7 based on previous event pattern in this subject.

### Dose limiting toxicity and MTD

Figure 1 illustrates the tolerance of C7 at 35% dose^19^ and at 45% dose as determined with the 3 + 3 design (group B). For group B, no DLT was observed at dose level 1 in age groups 1 and 3. As mentioned above, there was one DLT (grade 3 with intolerable vomiting and abdominal discomfort) in the first cohort of age group BII. No additional DLT was observed in the subsequent cohort enrolled in this group. Therefore, since 1/12 subjects experienced a DLT at dose level 1 (0/3 in group BI, 1/6 in group BII and 0/3 in group BIII), the MTD was defined as dose 1. De-escalation to dose 2 was not necessary.

### Safety

Analyses of all blood analytes revealed no significant change in the comprehensive metabolic panel, complete blood count, lipid panel, lactate, or β-hydroxybutyrate at the MTD (Tables 4 and 5).

**Table 4:** C7 safety in group B subjects.

Subject	Glucose (mg/L)		β-hydroxybutyrate (mM)		Lactate (mM)		Cholesterol (mg/dL)		Triglycerides (mg/dL)		Albumin (g/dL)	
	Pre C7	Post C7	Pre C7	Post C7	Pre C7	Post C7	Pre C7	Post C7	Pre C7	Post C7	Pre C7	Post C7
B1	84	81	0.1	0.1	1.9	1.7	118 L	129	35 L	50	3.3 L	3.2 L
B2	73	81	3.6	1.0	0.9	1.2	178	190	63	76	3.7	3.5 L
B3	82	101	0.8	0.1	0.8	1.4	146	152	47	105	3.5 L	3.4 L
B4	93	92	0.2	0.1	1.1	1.1	62	114	113	138	3.9	3.5 L
B5	83	80	0.1	0.2	1.3	1.1	174	143	117	170 H	3.7	3.4
B6	86	98	2.4	1.0	0.9	1.7	147	154	45	71	3.8	3.5 L
B7	79	88	1.8	0.3	1.4	0.7	216 H	179 H	81	83	3.9	3.8
B8	93	89	1.1	0.4	0.9	1.1	167	171	44	61	3.4 L	3.3 L
B9	92	87	0.6	0.2	1.3	1.8	141	127	51	58	3.5 L	3.3 L
B10	86	86	0.3	0.2	0.8	1.1	124 L	140	74	152 H	3.7	3.7
B11	82	96	0.1	0.1	1.1	2.4	142	144	44	89	4.0	4.1
B12	76	90	0.1	0.1	1.4	1.5	133	138	58	72	3.5 L	3.6

Fasting blood analytes immediately before and 10 days following C7 use in. *L:* Below reference range; *H*: Above reference range.

**Table 5:** C7 safety in group B subjects. Fasting hematological blood analytes immediately before and 10 days following C7 use. L: Below reference range.

Subject number	White blood count (× 1000/mm^3^)		Hemoglobin (g/dL)		Platelet count (× 1000/mm^3^)	
	Pre C7	Post C7	Pre C7	Post C7	Pre C7	Post C7
B1	8.6	11.9	12.0	11.6	341	323
B2	9.8	4.0 L	13.7	12.7	361	307
B3	7.4	5.9	14.3	13.8	359	348
B4	5.9	5.7	11.6 L	11.4 L	314	395
B5	5.2	5.4	14.2	13.4	235	232
B6	5.8	7.8	13.2	13.2	267	322
B7	4.5	6.4	15.4	14.1	287	298
B8	6.7	8.1	14.0	13.5	251	255
B9	11.1	7.0	11.8	11.7	405	368
B10	6.9	7.0	13.2	13.7	386	338
B11	6.4	5.5	13.8	15.0	268	342
B12	5.8	5.7	13.5	13.2	282	287

### C5 ketone body blood levels

Figure 2 illustrates the blood concentration of C5 ketone bodies following two doses of C7. The values are provided in Table 6. Although the purpose of these measurements was not to delineate the metabolokinetics of C7 derivatives, these results indicate that, while C5 ketosis was noted in all subjects, there was significant variability. In numerous cases there was no C5 ketosis measurable pre C7 treatment. In the rest of cases, where a measurable level of C5 ketones was detected before C7, the fold-change in ketone concentration in post-relative to pre-C7 conditions was 3- to 88-fold for BKP and 46-fold for BHP. No correlation was found between the levels of C5 ketones after C7 (R = 0.36), indicating that ketogenesis or circulating ketone body extraction is unequal for both ketone forms. There was also no correlation between pre-C7 β-hydroxybutyrate and post-C7 BKP levels (R = 0.32) or between pre-C7 β-hydroxybutyrate and post-C7 BHP levels (R = -0.5).

**Table 6:**
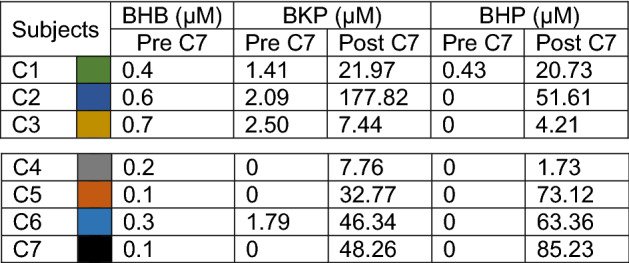
Blood C5 ketone body levels in group C subjects.

Values of C5 ketones before and after two oral doses of C7. Colors refer to subjects in Fig. 2. *BKP* β-ketopentanoate; *BHP* β-hydroxypentanoate; *BHB* β-hydroxybutyrate.

## Discussion

To enable the rigorous clinical investigation of C7 as a treatment for G1D, we have determined the MTD, safety and tolerability of C7 in G1D. The selection of C7 was based on its insipid nature, suitable for use as a dietary supplement, in conjunction with its heptanoate content. C7 dosing was based on individualized caloric consumption calculated at the time of study entry rather than per-weight or other dosing methods because caloric requirements change during development and C7 is fully consumed in the course of metabolism. This contrasts with the dosing of interventions (such as drugs) that primarily modify receptors or other targets whose action depends on molecule-to-molecule binding and are not consumed for biological action.

The β-oxidation of carbons 1–4 of heptanoate generates two molecules of acetyl coenzyme A and one molecule of propionyl coenzyme A derived from carbons 5–7. The latter can enter the TCA cycle through propionyl coenzyme A carboxylase. In this context, triheptanoin (C7) contains three esterified heptanoic acid molecules. We previously used ^13^C-nuclear magnetic resonance (NMR) spectroscopy^32^, gas chromatography-mass spectrometry (GC–MS) and liquid chromatography-mass spectrometry (LC–MS) to elucidate the metabolism of infused [5,6,7-^13^C_3_]heptanoate in the G1D mouse^3^. This work revealed enrichment in heptanoate-derived plasma glucose via neoglucogenesis and increased cerebral acetyl coenzyme A and glutamine concentrations consistent with metabolism of heptanoate and or derivative C5 (i.e., containing five carbons) ketones in glia.

Previous C7 use in G1D and other disorders was based on doses that supply up to 35% of the daily caloric intake and are tolerable^19–23^. However, augmentation of metabolic flux via increase substrate or preserved enzyme activity is desirable to maximize potential neural performance benefits. This is exemplified by the effect of increased blood glucose level or availability on neural function, including the amelioration of seizures, in G1D^7,33^. A parallel metabolic enzyme activity-neural function correlate is illustrated by the absence of detectable metabolic deficits in the brain of mice mildly deficient in pyruvate dehydrogenase^34^, which become pronounced under a greater degree of deficiency^4^. Thus, we aimed for as high as tolerable a dose, with the limitation that all oils, including the form in which C7 is manufactured, can cause gastrointestinal intolerance. A second limitation to greater doses stems from a universal desire to retain a diet composition as common as possible^35^ and from the necessity to additionally consume essential fat not provided by C7^27,28^.

Our subject selection from three age groups was based on age-dependent biological considerations and clinical observations to be considered in future clinical trials. First, nutritional fat requirements are age-dependent, varying by as much as 15% from early childhood to adolescence^27,28^. Second, the response to C7 might depend on age, which itself influences the level of fat consumption^36^ and on brain metabolic rate^37^. The cerebral metabolic rate for glucose is low at birth and rises to adult values by 2 years of age. It then continues to rise until, by 6 years, it nearly doubles. This elevated rate is maintained until approximately 10 years, when it begins to decline, approaching again the adult rate again near the end of the second decade. This time course parallels changes in the number of neurons, synapses, and dendritic spines in the human brain^38,39^.

The results indicate that the C7 MTD is not age dependent, as tolerability was similar for all ages. Consistent with the normal variability of stimulated blood ketone body levels in children^40^, the concentrations of the two C5 ketone bodies were variable across individuals and exhibited no correlation in several particular individuals. There was also no correlation between C5 ketogenesis and prior (i.e., immediately pre-C7) β-hydroxybutyrate ketogenesis. This suggests that ketogenesis and/ or blood ketone extraction arising from standard dietary fat and from C7 do not stem from the same metabolic process. Factors other than blood level determine metabolic efficacy (or lack thereof), especially considering the avid uptake of C5 ketones by several tissues rich in 3-oxoacid-CoA transferase, including the brain^41–43^. Correlating blood level with efficacy will be the subject of separate work. Of note, there are several possible metabolic effects derived from heptanoate, or its byproducts, in the brain^3^. This is due to the brain fuel potential of C5 ketones, heptanoate itself, and glucose from neoglucogenesis, all of which would be difficult to mechanistically separate without co-infusion labeling or other complex studies given the uncertainties about the magnitude of some of the relevant metabolic reactions^44^.

C7 is advantageous over other dietary therapeutic modalities given the possibility of combination with a regular or modified Atkins diet. This allows for a balanced diet, whereas the ketogenic diet is carbohydrate- and protein-restricted in favor of fat. Additionally, non-epileptic forms of G1D (i.e., apraxic, choreic, dystonic) continue to emerge^45,46^. Thus, simpler, effective diets are anticipated to be more widely accepted. Finally, relatively unbiased tools such a whole-exome or genome DNA analyses, comprehensive genomic hybridization and Sanger sequencing gene panels^47^ are increasingly uncovering G1D in young infants, for whom the ketogenic diet has not been fully tested^48,49^ but has received significant attention as a general epilepsy treatment^50^. Further, this diet provides reduced anaplerotic potential during this period of rapid brain growth^51^. Further studies will elucidate the compatibility of C7 with the ketogenic diet.

Our standard 3 + 3 phase I design^52,53^ yielded the MTD. Over 1,200 phase I oncology studies utilized this approach from 1991 to 2006^53^, with a large fraction of subsequent studies adhering to the same design^54^. To our knowledge, the 3 + 3 design has not been used with neurologic or pediatric drug or food supplement investigations. Our results suggest that, despite a variety of existing trial design alternatives, the conceptual and procedural simplicity of the 3 + 3 design allow for effective dose finding^53^, especially in disorders with low prevalence or when drug availability is limited.

## Conclusions

We were primarily motivated by the lack of an effective treatment for G1D. This includes, with few exceptions antiseizure medications^7^, the ketogenic diet and the modified Atkins diet^35,46^. C7 provides an anaplerotic alternative to these treatments. At 45% of caloric intake, C7 is safe and, after accounting for a period of frequent but transient gastrointestinal disturbance, suitable for clinical investigation. Future work may characterize C7 efficacy, including long term safety and tolerability, compatibility with the ketogenic diet and sources of individual variation in C5 ketone body metabolism.

## Supplementary Information

Supplementary Information.

## Data Availability

All the data are publicly available from the corresponding author.

## Abbreviations

BKP: β-Ketopentanoate
BHP: β-Hydroxypentanoate
C7: Triheptanoin
DLT: Dose limiting toxicity
G1D: Glut1 deficiency
Glut1: Glucose transporter protein type 1
PET: Positron emission tomography
