# Supplementary material for: Maximum dose, safety, tolerability and ketonemia after triheptanoin in glucose transporter type 1 deficiency (G1D)
Source: Sci Rep. 2023 Mar 1;13:3465. doi: 10.1038/s41598-023-30578-z (PMC9977760; doi:10.1038/s41598-023-30578-z)
Supplement: Supplementary file 1 — Supplementary Information. [file 41598_2023_30578_MOESM1_ESM.docx]

**Supplement**

**Maximum dose, safety, tolerability and ketonemia after**

**triheptanoin in glucose transporter type 1 deficiency (G1D)**

Ignacio Málaga ^1 *^, Adrian Avila^1 *^, Sharon Primeaux ^1^,

Raja Reddy Kallem ^2^, Charles R. Roe ^1, #^, William C. Putnam ^2, 3^,

Jason Y. Park ^4^, Shlomo Shinnar ^5^, Chul Ahn ^6^, Juan M. Pascual ^1, 7, 8, 9^

* Equal contribution. # Deceased

1 Rare Brain Disorders Program, Department of Neurology, The University of Texas Southwestern Medical Center, Dallas, Texas, USA; 2 Department of Pharmacy Practice and Clinical Pharmacology, Experimental Therapeutics Center, Texas Tech University Health Sciences Center, Dallas, Texas 75235, USA; 3 Department of Pharmaceutical Science, School of Pharmacy, Texas Tech University Health Sciences Center, Dallas, Texas 75235, USA. 4 Department of Pathology, The University of Texas Southwestern Medical Center, Dallas, Texas, USA. 5 Departments of Neurology and Pediatrics, Albert Einstein College of Medicine, Bronx, New York 10467, USA. 6 Department of Population and Data Sciences, The University of Texas Southwestern Medical Center, Dallas, Texas, USA; 7 Department of Physiology, 8 Department of Pediatrics, 9 Eugene McDermott Center for Human Growth & Development / Center for Human Genetics, The University of Texas Southwestern Medical Center, Dallas, Texas, USA

**Supplementary tables**

| Subject number | Age at enrollment (years), gender | Phenotype | *SLC2A1* variant | Previous treatments | C7 dose group |
| --- | --- | --- | --- | --- | --- |
| A1 | 2, M | Epilepsy, ID | c.997C>T  (p.Arg333Trp) | KD | Group AI |
| A2 | 4, F | Epilepsy, ID | PET positive | KD | Group AI |
| A3 | 10, M | Epilepsy, ataxia | c.653G>A  (p.Arg218His) | KD, MAD | Group AII |
| A4 | 9, F | Epilepsy, ID | Partial gene deletion including exon 3 and part of exon 4 | ASM | Group AII |
| A5 | 11, M | Epilepsy, ataxia, ID | c.18+1 G>A | ASM | Group AII |
| A6 | 10, M | Epilepsy | Exon 10 frameshift | KD, MAD | Group AII |
| A7 | 11, F | Epilepsy, ataxia | c.73C>T  (p.Gln25*) | KD | Group AII |
| A8 | 22, F | Epilepsy, ataxia | c.436G>A  (p.Glu146Lys) | ASM | Group AIII |
| A9 | 14, F | Epilepsy, ID | c.1454TC>T  (p.Pro485Leu) | KD, MAD | Group AIII |
| A10 | 27, M | Epilepsy, ataxia | c.377G>A  (p.Arg126His) | ASM | Group AIII |
| A11 | 14, M | Epilepsy | arr[GRCh37] 1p34.2(43387417_43431083)x1 | KD | Group AIII |
| A12 | 16, M | Epilepsy, ID | c.997C>T  (p.Arg333Trp) | KD, MAD | Group AIII |
| A13 | 20, M | Epilepsy, ataxia | c.972+1G>T  (splice site intron 7) | KD | Group AIII |
| A14 | 17, F | Epilepsy, ataxia | c.505_507delCTC  (p.Leu169del) | KD | Group AIII |

**Table S1**. **Group A subjects**. Demographic, clinical and genetic characteristics and previous treatments of G1D subjects treated with C7 at 35% of daily caloric intake. M: male, F: female, ID: intellectual disability, KD: Ketogenic diet, MAD: Modified Atkins diet, ASM: antiseizure medications, PET positive: diagnosed via fluorodeoxyglucose PET study.

| **Dose-finding study (group B)** |
| --- |
| **Inclusion criteria**:   - Ages between 2 years 6 six months to 35 years 11 months old, inclusive - Confirmed diagnosis of G1D either genetically or based on clinical, laboratory and characteristic brain ^18^fluorodeoxyglucose PET scan - No dietary treatment other than modified Atkin’s diet at enrollment - Analytical demonstration of normal general organ function and lipid profile.   **Exclusion criteria**:   - Previous life-threatening seizure episodes, including not limited to status epilepticus and cardiac arrest - Unrelated metabolic and/or genetic disease - Body mass index (BMI) greater than or equal to 30 - Chronic gastrointestinal disorder, such as irritable bowel syndrome, Crohn’s disease, or colitis - Use of a ketogenic diet, medium chain triglyceride (MCT)-supplemented diet, or low glycemic index diet - Treatment with C7 or MCT one month prior to enrollment - Pregnant or breast-feeding women - Active drug or alcohol use - Dementia or other progressive brain disorder |
| **Ketonemia study (group C)** |
| **Inclusion criteria:**   - Diagnosis of glucose transporter type I deficiency (G1D), confirmed by clinical genotyping at a CLIA-certified laboratory or by PET scan - Stability of diet, consuming either a modified Atkins diet or on no dietary therapy for at least one month - Age 24 months to 35 years old   **Exclusion criteria:**   - Unrelated metabolic or genetic disease - Chronic gastrointestinal disorder, such as irritable bowel syndrome, Crohn's disease, or colitis - BMI greater than or equal to 40 - Dietary therapy (i.e., ketogenic diet, MCT supplemented diets, Atkins diet, low glycemic index diet) - No evidence of abnormal EEG (spike wave discharges) in the previous 12 months - Women who were pregnant or breastfeeding or women planning to become pregnant during the course of the study - Allergy or sensitivity to C7 - Consumption of triheptanoin in the previous month - Dementia or other progressive brain disorder - Active drug or alcohol use - Inability or unwillingness of subject or legal guardian to provide written informed consent, or assent for children age 10-17 - Addition of a new antiseizure drug in the previous 3 months |

**Table S2**. Eligibility criteria for subjects in groups B and C.
